# The Incidence and Health Economic Burden of Ischemic Amputation in Minnesota, 2005-2008

**Published:** 2011-10-15

**Authors:** James M. Peacock, Hong H. Keo, Sue Duval, Iris Baumgartner, Niki C. Oldenburg, Michael R. Jaff, Timothy D. Henry, Xinhua Yu, Alan T. Hirsch

**Affiliations:** Center for Health Promotion, Health Promotion and Chronic Disease Division, Minnesota Department of Health; Division of Epidemiology and Community Health, University of Minnesota School of Public Health, and Minneapolis Heart Institute Foundation, Minneapolis, Minnesota, and Swiss Cardiovascular Center, Division of Angiology, University Hospital, Bern, Switzerland; Division of Epidemiology and Community Health, University of Minnesota School of Public Health, and Minneapolis Heart Institute Foundation, Minneapolis, Minnesota; Swiss Cardiovascular Center, Division of Angiology, University Hospital, Bern, Switzerland; Division of Epidemiology and Community Health, University of Minnesota School of Public Health, Minneapolis, Minnesota; Massachusetts General Hospital and Harvard Medical School, Boston, Massachusetts; Minneapolis Heart Institute Foundation, Minneapolis, Minnesota; Department of Epidemiology and Biostatistics, School of Public Health, University of Memphis, Memphis, Tennessee; Cardiovascular Division and Lillihei Heart Institute, University of Minnesota Medical School, Minneapolis, Minnesota

## Abstract

**Introduction:**

Critical limb ischemia (CLI) is the most severe manifestation of peripheral artery disease (PAD), is associated with high rates of myocardial infarction, stroke, and amputation, and has a high health economic cost. The objective of this study was to estimate the incidence of lower limb amputation, the most serious consequence of CLI, and to create a surveillance methodology for the incidence of ischemic amputation in Minnesota.

**Methods:**

We assessed the incidence of ischemic amputation using all inpatient hospital discharge claims in Minnesota from 2005 through 2008. We identified major and minor ischemic amputations via the International Classification of Diseases, 9th Revision, Clinical Modification (ICD-9-CM) procedure codes for lower limb amputation not due to trauma or cancer and assessed geographic and demographic differences in the incidence of ischemic amputation.

**Results:**

The age-adjusted annual incidence of lower limb ischemic amputation in Minnesota during the 4-year period was 20.0 per 100,000 (95% confidence interval, 19.4-20.6). Amputations increased significantly with age, were more common in men and in people with diabetes, and were slightly more common in rural residents. The number of amputation-related hospitalizations was steady over 4 years. The median total charge for each amputation was $32,129, and cumulative inpatient hospitalization charges were $56.5 million in 2008.

**Conclusion:**

The incidence of ischemic amputation is high and results in major illness and health economic costs. These data represent the first population-based estimate of ischemic amputation at the state level and provide a national model for state-based surveillance.

## Introduction

Lower extremity peripheral artery disease (PAD) is defined as blockages in the major arteries that supply the leg (1,2). PAD is 1 of the 3 major systemic atherosclerotic diseases and has a prevalence similar to that of coronary heart disease and ischemic stroke. People with PAD may be asymptomatic but often experience claudication as the primary ischemic symptom that causes exertional leg muscle pain. When atherosclerotic artery blockages in the leg progress and become severe or when a clot travels to completely block a leg artery (arterial thromboembolism), unremitting ischemia at rest can occur. This presentation of PAD is defined as critical limb ischemia (CLI) and signifies an imminent risk of amputation if revascularization is not achieved. The etiology of more than 80% of amputations that occur in all developed nations, including those that occur in people with diabetes, are due to PAD and CLI (3). The incidence of CLI will likely increase as the population ages because of the increasing prevalence of diabetes. If so, these preventable, morbid, and costly limb ischemic events will demand greater medical and revascularization resources (4). This burden has an effect at the family, city, and state levels.

Major lower extremity amputation occurs at an estimated rate of 120 to 500 per million per year in Western countries (5), and this rate increases with age (6). The rate is estimated at 25% in patients with CLI (1,2). Ischemic amputation is still a commonly performed operation, even in hospitals that emphasize aggressive revascularization for limb salvage, and occurs more often in the United States among blacks than whites (7). The age-adjusted annual rate of lower extremity amputation in the United States was reported to remain stable at approximately 30 per 100,000 per year from 1979 to 1989, despite an increase in the annual rate of percutaneous transluminal angioplasty and peripheral bypass surgery (8). Hallett et al reported a decrease in the rate of major (but not minor) lower extremity amputation (from 36.7 to 19.0 per 100,000 annually from 1973 to 1992), while the rate of percutaneous and operative revascularization increased during the same period (6). More recent data from Goodney et al suggest a decrease in major lower extremity amputation rates and lower extremity bypass surgery, with an associated increase in rates of endovascular procedures in older adults (9). These conflicting estimates use different case ascertainment strategies, and we are not aware of any effort in North America or Europe to conduct surveillance of ischemic amputation rates.

The aim of this investigation was to create a surveillance methodology for the incidence of ischemic amputation in Minnesota and to identify potential geographic and demographic disparities in incidence. This method was also designed to permit creation of a conservative estimate of the direct costs and health economic burden associated with ischemic amputation at the state level. We hypothesized that there would be heterogeneity in ischemic amputation rates on the basis of geographic regions (eg, urban vs rural); that ischemic amputation would represent a significant cardiovascular disease burden compared with both coronary heart disease (CHD) and stroke mortality; that hospital-related charges and costs would be comparable to those for CHD and stroke; and that trends in the incidence of amputation may be evident during a 4-year surveillance period. We intended to close a knowledge gap on ischemic amputation and describe a surveillance strategy that could be modeled by other states.

## Methods

We used data from a claims-based administrative data set compiled annually by the Minnesota Hospital Association, using the Uniform Billing Code of 1992 and the Uniform Billing Code of 2004 established by the National Uniform Billing Committee. This claims dataset contains information on inpatient hospitalizations at participating hospitals in Minnesota, including approximately 97% of the staffed inpatient beds at acute care hospitals. Information on Minnesota residents hospitalized outside the state was limited to the bordering states of North Dakota, South Dakota, and Iowa; information on hospitalizations in neighboring Wisconsin was not available. We used discharges that took place from January 1, 2005, through December 31, 2008. The dataset includes disease and procedure codes from the International Classification of Diseases, 9th Revision, Clinical Modification (ICD-9-CM), and patient sex, age, residential zip code, county of residence, date of admission, date of discharge, payer type, total dollar charges, and discharge disposition.

We identified lower limb amputations through ICD-9-CM codes. For each hospitalization record, up to 24 disease codes and 8 procedure codes are present. We first identified hospitalizations with any diabetes or cardiovascular disease code in the primary or secondary positions (ICD-9-CM 250.xx or 390.xx-459.xx), excluding patients with an injury code in the primary position (ICD-9-CM 800.xx-999.xx). We then identified lower limb ischemic amputations by ICD-9-CM procedure codes for lower extremity amputation (ICD-9-CM 84.10-84.19), excluding cases with an ICD-9-CM disease code for cancer (including malignant neoplasm of the bone, malignant neoplasm of skin, melanoma, and Kaposi's sarcoma). We further classified amputations as major (at or above the ankle) or minor (below the ankle). For comparison, we also identified CHD, stroke, and transient ischemic attack (TIA) hospitalizations by the presence of ICD-9-CM disease codes 410.xx-414.xx (CHD) and 430.xx-438.xx (stroke and TIA) in the primary position.

To estimate the total costs for each hospitalization, we adjusted total dollar charges by the hospital-specific cost-to-charge ratio at the time of patient discharge. For hospitalizations at facilities with no cost-to-charge ratio, depending on the hospital's location, we used the average rural hospital or urban hospital ratio for the state. Hospitalizations in South Dakota (n = 69) were not identified by facility, so we substituted the urban hospital cost-to-charge ratio, on the basis of known patterns of care for Minnesota residents hospitalized in that state. We applied this adjustment to estimate the total resource cost associated with inpatient care only, as it does not reflect costs associated with out-of-hospital imaging, procedures, office-based care, outpatient rehabilitation, or use of limb prostheses or chronic medications (10-17). To account for price inflation, we adjusted all cost values using the US Bureau of Labor Statistics Consumer Price Index for Inpatient Hospital Services (18).

We calculated the population at risk by county, sex, age group (0-4 y, 5-14 y, 15-24 y, 25-34 y, 35-44 y, 45-54 y, 55-64 y, 65-74 y, 75-84 y, and ≥85 y), and year of discharge using vintage 2008 bridged-race postcensus population estimates of Minnesota supplied by the US Census Bureau and National Center for Health Statistics (19). The age-adjusted incidence of lower limb ischemic amputation was calculated using these annual population estimates and the number of hospitalizations during 4 consecutive years. We suppressed counts for counties with fewer than 11 total cases.

We defined urban residence as living in counties that were part of Metropolitan Statistical Areas as defined by the US Census Bureau in 2007; all other counties were classified as rural (20). The urban counties included approximately 72% of the total state population (approximately 5.2 million) from 2005 through 2008.

Continuous data are presented as medians (interquartile range); categorical data are presented as frequencies. Both inpatient hospitalization charges and costs are presented using medians, means, and sums. All analyses were conducted using SAS software version 9.2 (SAS Institute, Inc, Cary, North Carolina).

## Results

There were 4,302 hospitalizations for lower limb amputations among Minnesotans from 2005 through 2008 (Table 1). Of these, 1,831 (43%) were major, 2,470 (57%) were minor, and 1 was unspecified. The median patient age was 67, and major amputations were more likely to occur in older people. Most amputations took place among men and people who lived in an urban county. Of total amputations, 72% of cases were performed in people with diabetes, most of whom had a minor amputation.

The age-adjusted annual incidence of lower limb ischemic amputation in Minnesota during the 4-year period was 20.0 per 100,000 (95% confidence interval [CI], 19.4-20.6), which is more than half the annual stroke mortality (38.3 per 100,000; 95% CI, 37.5-39.1) and comparable to mortality for both diabetes (20.8 per 100,000; 95% CI, 20.2-21.4) and Alzheimer's disease (21.6 per 100,000; 95% CI, 21.0-22.2) (21). The figure illustrates the lower limb ischemic amputation incidence, CHD mortality rate, and stroke mortality rates by county in Minnesota from 2005 through 2008. Counties in the highest quartile of age-adjusted ischemic amputation incidence are concentrated in the northern half of the state, along with groups of counties in the southwest and southeast; all but 2 are rural. The annual incidence of ischemic amputation in rural counties is 21.4 per 100,000 (95% CI, 20.3-22.5), approximately 9% higher than in urban counties (19.6 per 100,000, 95% CI, 18.8-20.3). The overall pattern is similar to that for CHD mortality and to a lesser degree similar to that for stroke mortality.

**Figure.**
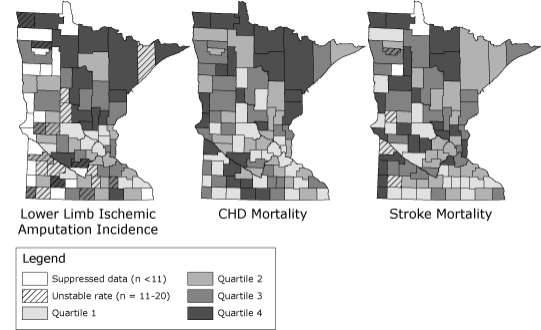
Lower limb ischemic amputations, coronary heart disease (CHD) mortality, and stroke mortality per 100,000 by Minnesota county, 2005-2008. Data for lower limb ischemic amputations obtained from Minnesota Hospital Uniform Billing Claims Data; Health Economics Program, Minnesota Department of Health; and the Minnesota Hospital Association. Data for CHD mortality obtained from Minnesota Department of Health Center for Health Statistics and International Classification of Diseases, 10th revision (ICD-10) codes I00-I09, I11, I13,and I20-I51. Data for stroke mortality obtained from Minnesota Department of Health Center for Health Statistics and ICD-10 codes I60-I69.

**Table:** 

Three maps depict the state of Minnesota with county borders. The first map displays the incidence of lower limb ischemic amputation; the second, coronary heart disease (CHD) mortality; and the third, stroke mortality.
The legend shows the quartile ranking of the outcome displayed on each map; quartile 1 represents the lowest incidence and quartile 4 represents the highest incidence. Data for counties with fewer than 10 cases over 4 years are suppressed, and data for counties with 11 to 20 cases over 4 years are shown but are noted as unstable.
In general, the highest lower limb ischemic amputation incidence and highest CHD and stroke mortality rates are concentrated in counties in the northern half of Minnesota and in rural counties. The pattern is similar, but not entirely overlapping, for all 3 outcomes.

The number of CHD hospitalizations declined during the study period, while the number of ischemic amputations and stroke hospitalizations were steady (Table 2). The median charge for ischemic amputation hospitalizations was similar to that for CHD and about twice as high as for stroke and TIA hospitalizations. The total direct inpatient hospital charges for all ischemic amputation hospitalizations approached 20% of the total charges for stroke and TIA.

The inflation-adjusted estimated median cost for ischemic amputation remained stable from 2005 through 2008, but the mean cost per hospitalization increased by 9.2% (Table 3). Compared with 2005, the number of annual events in 2008 was 11.5% lower, and total annual inflation-adjusted inpatient hospital costs were 3.8% lower. The pattern in the intervening years was not monotonic.

## Discussion

We used a representative hospital discharge data set from 2005 through 2008 to evaluate the incidence of ischemic amputation in Minnesota. These data demonstrated that most patients who underwent ischemic amputation were male and older. The age-adjusted annual incidence of ischemic amputation in Minnesota was low compared with the incidence reported elsewhere (6,8). Diabetes was prevalent in people who underwent ischemic amputation; 72% of the total ischemic amputation population had diabetes, and 63% of patients who had a major amputation had diabetes. Other studies have reported a prevalence of diabetes in ischemic amputees that ranges from 45% to 61% (22,23). Furthermore, we found that the resource allocation by hospitals and economic burden was high; people who underwent ischemic amputation had long hospital stays. Median inpatient hospitalization charges were similar to those for CHD patients and double those for stroke and TIA patients.

Though the systemic atherosclerosis disease process provides a common etiology for stroke, myocardial infarction, and PAD, and though systemic risk is elevated for people with each of these syndromes, the incidence of amputation, CHD mortality, and stroke mortality were not uniform across Minnesota. This finding likely reflects differing care standards for each condition, because reporting for each condition is likely to be similar (each outcome represents a "hard ischemic endpoint"). These data do not provide insight into distinctions in the process of care between coronary and stroke or CLI patients that may clarify these varying rates. Our results underscore — on a state-based and county-based level — the health disparity in care that affects the CLI population.

Visser et al estimated a cost of $45,200 for a major amputation in the first year, followed by an annual cost of $11,000 beyond the initial hospitalization (24). In a more selected population of patients treated for CLI, the immediate cost directly attributed to amputation was $40,000 (1). The addition of subsequent rehabilitation is usually associated with a doubling of these costs (1). Our cost findings in a more general population provide additional information about immediate hospitalization charges associated with ischemic amputation. The Healthcare Cost and Utilization Project Nationwide Inpatient Sample of 39.5 million discharges in the United States in 2007 estimated median charges for amputation due to "circulatory system disorders" (exclusive of the upper limb and toe) of $45,900 and median hospital costs of $15,900 (25). This value compares well with our estimates.

Minnesota is home to less than 2% of the total US population (19), and Minnesotans experience lower rates of atherosclerotic disease and lower medical costs than the national average (26-28). Nevertheless, our results delineate the health cost implications in the context of nationally limited health care resources. Ziegler-Graham et al estimated the prevalence of limb loss in the United States, projected these values through the year 2030, and suggested that ischemic limb loss is likely to rise with the aging population and an increase in diabetes and "dysvascular" disease (29).

Differences in ischemic amputation rates in the literature may be due to the unique populations included in those distinct datasets, differing case identification criteria for ischemic amputation, inclusion or exclusion of minor amputations or people with diabetes, inclusion of first amputation events versus cumulative per-patient event rates, or multiple amputations in the ipsilateral limb. Only through use of a consistent methodology will patients, payers, departments of health, and other agencies be able to make state-to-state comparisons. Consistent application of a common cross-state case ascertainment strategy would foster creation of estimates of the incidence of lower limb ischemic amputation and costs, permitting future comparison of population-based treatment strategies. Although racial and ethnic disparities in amputation rates have been elucidated, disparities based on geography, sex, and health care coverage may become visible from regional data. Health disparities, once revealed, provide an opportunity to implement preventive health strategies that can reduce high ischemic amputation rates across and within states.

Despite the use of a representative hospital discharge data set, this study had several limitations. The limited availability of clinical covariates in public data sets is acknowledged in the literature (30). Multiple hospitalizations for amputation (of the same or different limb) cannot be identified because of the absence of unique patient identifiers. Another limitation is that inpatient hospitalization charges, and the subsequent adjustment with cost-to-charge ratios, do not optimally reflect the resources expended in managing this condition. These data do not distinguish between use of health care resources that were expended to treat the episode of CLI versus any associated illnesses that existed during the index hospitalization. Additionally, these charges only reflect resources expended during the inpatient hospitalization and do not reflect costs after discharge. Our cost estimate is conservative, as it does not reflect any ongoing disability costs associated with ischemic amputation. Furthermore, data for counties with few cases of ischemic amputation (ie, 11-20) were reported, but the estimates are potentially unstable and should be interpreted with caution. Finally, our results were derived from a state that is known to be characterized by lower racial and ethnic diversity than the nation. Therefore, these data are not presumed to be generalizable to other states or regions. Despite these limitations, these data provide insight into the incidence and cost of ischemic amputation in Minnesota.

In conclusion, these data provide the first contemporary population-based estimate of ischemic amputation and hospitalization charges in a nonselected state-based population. Although the incidence of ischemic amputation in Minnesota is lower than has been reported from other studies, the direct inpatient hospital charges and estimated costs are high. The incidence of ischemic amputation was higher in rural counties. Ischemic amputation represents a significant statewide cardiovascular disease burden compared with both CHD and stroke mortality. This methodology could underpin an ischemic amputation surveillance strategy that could be modeled by other states nationally.

## Figures and Tables

**Table 1:** Population Characteristics of Ischemic Amputations in Minnesota, 2005-2008^a^

**Characteristic**	All Amputations (n = 4,302)	Minor Amputations (n = 2,470)	Major Amputations (n = 1,831)
Age, median (IQR), y	67 (56-79)	65 (54-76)	70 (59-81)
Male sex, %	65.4	67.5	62.5
Urban county residence, %	65.1	66.0	63.9
Diabetes, %	72.3	79.4	62.8
Length of stay, median (IQR), d	7 (4-12)	6 (4-10)	9 (6-14)
Inpatient charges, median (IQR), $	32,129 (17,980-57,761)	27,377 (16,087-47,737)	39,512 (21,414-73,174)
Inpatient hospitalization costs, median (IQR), $	12,434 (7,402-21,714)	10,609 (6,525-18,127)	15,246 (8,992-26,912)

Abbreviation: IQR, interquartile range.

a A minor amputation is any amputation below the ankle, and a major amputation is any amputation at or above the ankle; 1 amputation was at an unspecified site.

**Table 2:** Inflation-Adjusted Direct Inpatient Hospital Charges for Ischemic Amputation, Coronary Heart Disease, and Stroke in Minnesota, 2005-2008^a^

**Condition**	2005	2006	2007	2008
**Ischemic amputation**				
Mean, $	51,952	55,521	55,527	55,311
Median, $	35,069	37,456	33,722	35,893
Total, $	59,849,292	61,684,119	56,470,502	56,528,330
Hospitalizations, n	1,152	1,111	1,017	1,022
**Coronary heart disease^b^**				
Mean, $	43,280	40,412	41,924	40,999
Median, $	34,954	33,027	34,213	33,321
Total, $	1,108,671,561	991,833,792	924,145,457	876,268,816
Hospitalizations, n	25,616	24,543	22,043	21,373
**Stroke and transient ischemic attack^c^**				
Mean, $	29,008	27,706	29,361	29,709
Median, $	18,173	16,917	17,921	17,831
Total, $	351,641,640	336,098,550	338,327,622	349,819,464
Hospitalizations, n	12,122	12,131	11,523	11,775

Abbreviation: ICD-9-CM, International Classification of Diseases, 9th Revision, Clinical Modification.

a All charges are inflated to 2008 $US using the Consumer Price Index for inpatient hospital care.

b Coronary heart disease hospitalizations, primary ICD-9-CM disease code, 410-414.

c Stroke and transient ischemic attack hospitalizations, primary ICD-9-CM disease code, 430-438.

**Table 3:** Inflation-Adjusted Inpatient Hospital Costs for Ischemic Amputation in Minnesota, 2005-2008^a^

**Estimated Inpatient Hospital Cost**	2005	2006	2007	2008
Mean, $	19,230	21,163	20,790	21,015
Median, $	13,852	14,873	12,829	13,466
Total, $	22,240,233	23,469,227	21,102,517	21,393,668
Hospitalizations with estimable costs, n	1,150	1,109	1,015	1,018

a Costs calculated from hospital-specific cost-to-charge ratios in effect at time of patient discharge. All cost values are inflated to 2008 $US using the Consumer Price Index for inpatient hospital care.
